# Are the doctors of the future ready to support breastfeeding? A cross-sectional study in the UK

**DOI:** 10.1186/s13006-020-00290-z

**Published:** 2020-05-20

**Authors:** Kirsty V. Biggs, Katy J. Fidler, Natalie S. Shenker, Heather Brown

**Affiliations:** 1 Royal Stoke University Hospital, Newcastle Road, Stoke-on-Trent, ST4 6QG UK; 2 Brighton and Sussex Medical School, Falmer, BN1 9PX UK; 3 Department of Surgery and Cancer, Imperial College London, IRDB Hammersmith Hospital, London, W12 0HS UK

**Keywords:** Medical education, Breastfeeding, Clinical skills, Medical training

## Abstract

**Background:**

Currently there is no published data on the inclusion of breastfeeding education within the UK medical school curriculum. This study aims to address this knowledge gap and explore students’ perceptions of their readiness to support breastfeeding.

**Methods:**

An online survey was used to collect data from 32 UK undergraduate medical schools and their students. All students in their final two years of study at the 30 universities offering a 5- or 6-year medicine course, were eligible.

**Results:**

Curriculum data was obtained from 26 (81%) institutions. Compulsory breastfeeding education was provided by 85% (*N* = 22) institutions with 81% (*n* = 21) providing lecture-based teaching and 19% (*n* = 5) offering formal clinical education. Overall, 411 students from 22 institutions participated. A moderate ability to identify the benefits of breastfeeding was observed; however, self-rated confidence in practical skills was poor. Assisting with latching was the least confident skill, with confidence in only 3% (14/411) students. Most students (93%) viewed doctors as playing an important role in breastfeeding, with those interested in either women’s health, paediatrics or general practice perceiving the role of doctors as more important. Overall, 93% (381/411) students requested further breastfeeding education.

**Conclusions:**

This study suggests UK medical schools are not adequately preparing students to support breastfeeding patients. Further studies should explore the competency of doctors to meet the needs of lactating women, and design optimal training for UK medical students.

## Background

Over the course of human evolution, breastfeeding has played a pivotal role, with established benefits for the short- and long-term health of the mother and child [1, 2]. Far from simple nutrition, breastmilk is a complex bioactive fluid and developmental patterning tool, affecting cognition, immune maturation and physiology [3]. The World Health Organization recommendations are that infants should be exclusively breastfed for around 6 months, continuing for two years and beyond, as long as mother and baby wish. In the UK, less than 1% of mothers are breastfeeding by the time their baby is one year [4].

The Unicef Baby Friendly Initiative is a global endeavour that seeks to entrench breastfeeding as a public health target, explicitly stating that all healthcare staff should be trained in the necessary skills to support breastfeeding [5].

Clinicians can be highly influential figures when parents are making decisions about infant feeding; however international data from a Cochrane review suggests that doctors rarely receive appropriate training to support and manage issues related to breastfeeding [6, 7]. In the UK, there is no standardised breastfeeding education for clinicians [8, 9]. This lack of education is also reflected overseas with less than 10% of general practitioners (GP) in an Irish study [10], and less than 50% of GP trainees in Australia receiving formal breastfeeding training [11].

There are several examples in the literature of where poor knowledge and training around infant feeding has been shown to have negative impacts. In a Norwegian survey of GPs, only 26% could identify the differences between infant formula and human milk, which consequently affected their confidence in counselling patients [12]. Furthermore, approximately half of GPs and paediatricians surveyed in the UK advised parents to introduce solids before the WHO/UNICEF recommendation of around 6 months [13]. Mothers who were inappropriately recommended by paediatricians to supplement with infant formula are more likely to have discontinued breastfeeding by 12 weeks [14].

Medical school builds the foundation for all doctors’ clinical practice and is a perfect opportunity to educate future clinicians with the essential skills and knowledge to support their patients [15, 16]. Furthermore, it could be argued that regardless of which specialty pathway the medical graduate follows, they are likely to have contact with breastfeeding patients during their hospital and community training. Currently there is no published data on infant feeding education provided within undergraduate medicine courses in the UK. International surveys suggest that medical students are not well enough equipped with the counselling skills and knowledge to support breastfeeding mothers [17–19].

This study aimed to collect data on the inclusion of breastfeeding education within the undergraduate curriculum at UK medical schools and to explore student perceptions of their role as future doctors in supporting breastfeeding.

## Methods

### Study design

A cross-sectional study was conducted to collect data on the delivery of teaching around breastfeeding within the undergraduate medical school curriculum and explore medical student perceptions on breastfeeding education using two separate online survey tools.

### Medical school recruitment

Within the UK, 34 medical schools provide undergraduate teaching. Two medical schools are exclusively for postgraduate students and were excluded from our study. Of the 32 remaining, 30 provide a 5- or 6-year complete curriculum. The remaining two provide only 2 years of preclinical teaching. All 32 medical schools were invited through email to partake in an online survey on the inclusion of breastfeeding education within their undergraduate curriculum. Medical school curriculum support staff were emailed with a brief background, inviting them to participate in an online questionnaire. Consent was confirmed by participation and the right to withdraw was explained. Data for the student surveys was analysed only for the 30 medical schools with a full 5 or 6 year curriculum.

### Medical student recruitment

All undergraduate students in their final 2 years of study at the 30 UK medical schools offering a 5- or 6-year medicine course were eligible for participation in an online medical student survey. Gatekeeper approval was sought from each eligible medical school to gain consent before students were contacted. A standardised message was sent to university staff and internal ethics applications were made depending on the framework of the medical school.

A standardised message, participant information statement and link to the online survey was either sent via student email system or advertised on the relevant intranet site of the university depending on the institution’s policy (Additional File 1). Students were provided with the aims of the research and information regarding their voluntary participation, right to withdraw and the anonymity of the results. By participating, the students consented to the study.

### Materials

Two online questionnaires were designed, one for assessing breastfeeding education provided within the curriculum and the other to determine their students’ knowledge and perceptions (Additional File 2). Data was collected from curriculum staff relating to the type of education offered and in which year groups and modules this was delivered. Student questionnaires collected data on demographics, their confidence in supporting breastfeeding mothers, the perceived role of doctors in breastfeeding support, career aspirations, and interest in receiving additional teaching on breastfeeding. Questions included multiple choice, ranked answers and Likert scales.

### Data analysis

Microsoft Excel was used for the collation of basic percentage data. IBM SPSS 24 statistical software was used for all other statistical analysis. For categorical data Pearson’s Chi-squared test was used to determine significant differences between groups. For non-parametric data, the Mann-Whitney U Test was used. *P*-values < 0.05 were considered significant. Histograms were used to determine the data distribution.

## Results

### Participants

In total, 26 out of 32 eligible medical schools completed the initial online curriculum survey between March 2017 and September 2017. 25 out of 30 eligible medical schools consented for their students to be surveyed, with participation from medical students at 22 out of 25 consented universities. Overall, 411 medical students in their penultimate and final years of study participated in the online survey between March 2017 and March 2018. There was a median of 13 (3.25, 27) student participants per medical school.

### Medical school education

Curriculum data on 81% (26/32) of the eligible UK undergraduate medical schools was collected with regards to breastfeeding education (Table 1). 85% (22/26) of participating medical schools provided compulsory breastfeeding teaching (with the requirement that all students attend), three provided optional teaching and one did not include any breastfeeding education in their curriculum. Over three-quarters (81%) of the medical schools provided lectures on breastfeeding, with around two-fifths (46%) delivering seminars or small group teaching. Lectures were often provided in a pre-clinical context focusing on lactation physiology, whereas clinical exposure was usually with the postnatal midwifery team. Often this was unstructured and delivered on an ad hoc basis. Formal clinical teaching on breastfeeding is provided at five (19%) medical schools as part of general practice, paediatrics and obstetric modules. Details of the different teaching methods are available in the supplementary materials (Additional File 3).

**Table 1:** Medical school curriculum

Overview	*n* (%)
No. universities consenting to curriculum survey	26/32 (81)
Provide compulsory breastfeeding education	22 (85)
Lecture-based teaching	21 (81)
Seminars/ small group teaching	12 (46)
Formal clinical teaching	5/26 (19)
Ad hoc clinical teaching/ experience	15/26 (58)

### Medical student characteristics

Table 2 presents the demographic data from the 411 student participants in our online survey. Over two-thirds of the students were female (72%, *n* = 295) and 82% (*n* = 336) were interested in a career in either obstetrics and gynaecology, paediatrics or general practice. 84% (*n* = 347) of our medical student responders were aged between 21 and 25 years old.

**Table 2:** Medical student characteristics (*n* = 411)

Demographic details	*n* (%)
Male	116 (28)
Female	295 (72)
Age (years)	
21–25	347 (84)
26–30	46 (11)
31–35	9 (2)
> 36	9 (2)
Had a career interest in obstetrics and gynaecology, paediatrics and/or general practice	336 (82)
Obstetrics and gynaecology	148
Paediatrics	160
General practice	244
Number of students with career interests in more than one of the given specialities	117

### Breastfeeding knowledge of medical students

In order to assess knowledge of the benefits of breastfeeding, medical students were asked to select from a list of given statements (Additional File 4). Overall, 92% were able to successfully recognise that breastmilk contains antibodies and hormones. Other correct options identified from the list included: emotional attachment (97%), reduced infantile infections (90%), reduced maternal risk of breast and ovarian cancer (78%), reduced risk of obesity and type II diabetes mellitus in adulthood (77%), reduced risk of necrotising enterocolitis (69%) and reduced environmental impact (62%).

### Medical student clinical confidence

**Fig. 1:**
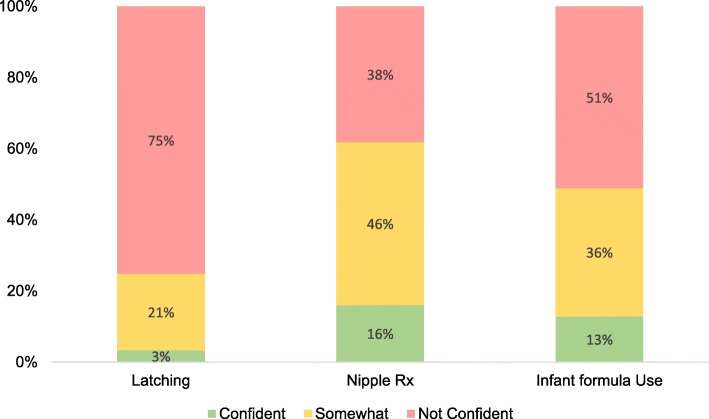
Medical student clinical confidence in performing breastfeeding related skills. Latching; confidence in helping a mother with latching problems; Nipple Rx: confidence in recognising and managing mastitis and nipple thrush; Infant formula use: confidence on advising on medical reasons for supplementing breastfed infants with formula

Students were asked to self-assess their confidence in performing breastfeeding related clinical skills (Fig. 1). Across the three skills, a minority of medical students assessed themselves as confident. 16% (*n* = 66) felt confident at recognising and managing mastitis and nipple thrush, 13% (*n* = 53) were confident they could advise on medical reasons for supplementing breastfed infants with formula, and only 3% (*n* = 14) were confident in assisting with latching. Receiving compulsory breastfeeding teaching was not significantly associated with students’ self-rated skills performance, however students who had received formal clinical teaching (*p* = 0.04) or small group teaching (*p* < 0.01) had significantly more confidence at diagnosing and managing nipple problems (Additional File 5).

### Doctors’ role in breastfeeding

Medical students were asked to rank six categories of influencer with respect to how influential they are upon infant feeding decisions of mothers (Table 3). Partners were ranked as most influential with the doctor ranked fourth out of six. Student career aspirations had no effect upon the mean ranked score (Additional File 6). Overall, 44% thought doctors played a very important role in breastfeeding support, 49% viewed it as quite important, and only 7% thought it was not within the remit of the doctor. Medical students who had a career interest in either obstetrics and gynaecology, paediatrics or general practice perceived the doctors’ role in breastfeeding support as more important (*p* = 0.01; Additional File 7). Student gender and teaching received at medical school were not statistically associated with their perceived importance of supporting breastfeeding as a clinician.

**Table 3:** Medical students’ ranked perceptions of which figure is most influential upon a mother’s infant feeding decisions

	Mean ranking (1–6)
Partner	2.39
Midwife	2.6
Mother	3.01
Doctor	3.76
Other family member	4.4
Other healthcare professional	4.8

1: most influential; 6: least influential

### Medical student attitudes towards further breastfeeding education

Overall, 93% (381/411) of students surveyed stated they would like more training on breastfeeding within the undergraduate medical school curriculum. 9% (*n* = 26) required lecture-based teaching, 19% (*n* = 78) preferred practical skills training, whereas the most favoured teaching approach was a combined approach (65%, *n* = 267).

There were no statistical differences in student desire to receive further breastfeeding education with regards to their self-assessed skill confidence. However, of the students requesting teaching by combined methods, there was lower confidence in nipple management (*p* < 0.01) and formula use (*p* = 0.01, Additional File 8).

## Discussion

### The medical school curriculum

This is the first study to date looking at the inclusion of breastfeeding education within the undergraduate medical school curriculum in the UK. Currently, there is no standardised approach to the content and structure of the teaching provided, with 15% of the participating institutions providing no compulsory breastfeeding education. These findings were also reflected in the USA [20, 21]. An in-depth analysis of the curriculum at the United States Medical Institution revealed training deficits in both knowledge and skills-based competencies, which was reflected in their students’ self-rated breastfeeding knowledge and counselling abilities [22]. Furthermore, insufficient inclusion of breastfeeding within the undergraduate curricula has also been reported in Korea, with a lack of interactive learning opportunities and clinical exposure [23].

The Academy of Breastfeeding Medicine (ABM), a global organisation of physicians, produced an education statement on the learning objectives related to human lactation [24]. In line with the Baby Friendly Initiative, medical schools should aim to educate their students in both the theory and practice of breastfeeding. According to ABM, it is recommended that all students receive clinical training in the management of breastfeeding. Relevant skills to be covered included safe prescribing for breastfeeding, the facilitation of latching, counselling mothers and managing common problems [24]. Our study found that only 19% of institutions provided formal clinical exposure or training on breastfeeding.

### Are medical students ready to support breastfeeding?

In this study, we collected data on the preparation of medical students for supporting breastfeeding mothers. When provided with a list of true/false statements, students performed well at identifying the benefits of breastfeeding (Additional File 4). This was often covered in the lecture-based teaching, provided by most medical schools. Although students felt prepared to identify benefits of breastfeeding, their confidence to perform breastfeeding skills was low.. This is concordant with a systematic review of healthcare students demonstrating insufficient breastfeeding knowledge regarding assessment and management of breastfeeding-related issues [25]. A study in the USA found 100% nursing students were able to identify breastmilk as the optimum feeding choice, however 65% were unable to identify cracked nipples as a potential indication poor latching, with 62% believing cracked nipples are managed by expressing milk and refraining from breastfeeding for 24 h [26].

The General Medical Council’s ‘Outcomes for Graduates’ states that new doctors must be competent in providing explanation, advice and support to address their patients’ needs [27]. The students in our study recognised the role of the doctor in supporting breastfeeding. This was particularly the case in students with a career interest in either obstetrics and gynaecology, GP or paediatrics. However, according to the Educational Objectives and Skills for the Physician with Respect to Breastfeeding, outlined by ABM, “All physicians, regardless of discipline, should have basic knowledge and skills in breastfeeding maintenance, diagnosis, and treatment of related problems” [24].

Although students did not perceive the doctor to be the most influential figure in infant feeding decisions, their role in promoting breastfeeding was recognised. This view is supported in a study by Odom et al., with women naming the infant’s father, the maternal grandmother, and doctor as important figures [28]. Despite this, healthcare professionals may often underestimate their impact on such decisions. In a Scottish study, NHS staff felt their contribution to breastfeeding support were diminished due to external factors, with family members playing a much greater role, than the professional, in infant feeding decisions [29].

### Improving breastfeeding education

The majority (93%) of the students participating in our study stated they would like more teaching on breastfeeding at medical school. The perceived value of including undergraduate breastfeeding education is not a new viewpoint. A survey from the 1970s suggesting half of paediatricians and obstetricians would have benefited from a specific breastfeeding curriculum whilst at medical school [30]. This view is still relevant today with 85% Norwegian GPs believing that infant feeding should be taught before gaining their registration [12]. Additionally, doctors who reported lower confidence in breastfeeding clinical skills were less likely to engage with further training, suggesting the need to incorporate breastfeeding into a core curriculum [13].

The introduction of breastfeeding education is well-received by staff and students [31]. A medical school in New Mexico developed a module to include interactive teaching and patient visits, delivered via a multi-specialty approach [21]. In another example, 88% of medical students in India showed awareness of the BFI ‘ten steps of successful breastfeeding’ which was taught as part of the core curriculum and demonstrated successful integration into medical education [19]. A study of Brazilian healthcare students and professors have however identified the potential impracticalities of integrating breastfeeding into the already full-time undergraduate curriculum, with particular stress on the requirement for more practical classes on breastfeeding [32].

The medical students in our sample desired an integrated learning approach to include both theoretical knowledge and practical skills. The value of this teaching style is emphasised in the Carnegie Foundation for the Advancement of Teaching report [33]. A breastfeeding toolkit was developed with theoretical material for nursing students, however there was no statistical improvement in knowledge [34]. In addition, Taiwanese nursing students felt their theoretical training insufficiently prepared them to support mothers [35].

A recent Indonesian study demonstrated greater knowledge and counselling skills in final year students who had participated in an integrated module including hands-on teaching [18]. The successful implementation of breastfeeding roleplay intervention for healthcare volunteers was also demonstrated in Iran, with greater persisting knowledge and transfer of emotional experiences when compared to lecture-based education [36]. Our results support this finding with students who had received formal clinical teaching and/or small group sessions describing greater confidence in managing nipple pathology.

In the USA, a high-fidelity lactation simulation model was developed for educating healthcare professionals. During a development trial, physician residents reported benefits of being able to practice comfortable hand positioning during the breast assessment. Breastfeeding medicine physicians and midwifery students evaluated the final model as having realistic representations of lactation-related pathology and an effective breast pump mechanism to simulate hand expressing [37].

A recent scoping review of application-based breastfeeding education for nurses and physicians demonstrated a variety of teaching methods using real patients and roleplay, but found no standardised method of educational intervention or evaluation [38]. Several teaching methods have been suggested to provide an integrated breastfeeding education for the undergraduate medical student. These include online components, problem-based learning (PBL) and Objective Structured Clinical Examinations (OSCEs) [23]. A case-study in Lebanon has also identified the role of social networking in breastfeeding education to promote medical students self-efficacy and knowledge [39].

As there is a clear association between examinations and students’ learning, the ABM guidance stresses the additional importance of including breastfeeding in examinations to ensure students dedicate their efforts at learning the relevant material [40, 41].

### Limitations

The main limitation of this study was the inability to collect data from all UK undergraduate medical schools due to institutional permissions. The curriculum survey was completed by an allocated member of university staff; however, the response given may have varied depending on their role within the university. The majority of medical student respondents were female (Table 2, 72%), however this does reflect the national 3:2 ratio of female to male students in UK medical schools [42]. A recent study of GP registrars found that males doctors presumed that women with BF problems would want to see a female GP [11], which may infer that there is a perception among male trainees that this field is not relevant to them. Given the potential for all doctors to encounter breastfeeding mothers and influence infant feeding decisions, further work is needed to ensure skills that support the establishment and maintenance of breastfeeding engage all trainees.

## Conclusions

The current level of breastfeeding education within the curriculum at UK undergraduate medical schools is insufficient, with few able to ensure that their students gain adequate clinical exposure. This may be related to either lack of time or lack of priority in the curriculum. From this study and the literature, doctors play an important role in supporting breastfeeding, but medical students in their penultimate years of study do not feel adequately prepared to fulfil this role. Further studies should explore the competency of doctors to meet the needs of lactating women, and design optimal training for UK medical students. Our results and the existing literature suggest that an integrated core curriculum to include practical training is both necessary and imperative. With the advent of relatively cheap breastfeeding simulation for teaching systems this should now be feasible in most medical schools.

## Supplementary information

**Additional file 1.** Patient Information Sheet. Word document.

**Additional file 2.** Survey questions on SurveyMonkey. Word document.

**Additional file 3.** Medical school curriculum details. Word document.

**Additional file 4.** Table of medical students’ ability to correctly identify the benefits of breastfeeding (Table). Word document.

**Additional file 5.** Medical students self-rated confidence in clinical skills in relation to teaching provided by medical school (Table). Word document.

**Additional file 6.** Ranking of influential figures according to interest in obstetrics and gynaecology/paediatrics/general practice (Table). Word document.

**Additional file 7.** Influences upon medical students’ perceived importance of the doctor’s role in Breastfeeding support (Table). Word document.

**Additional file 8.** Interest in further teaching in medical schools according to medical students’ confidence at performing breastfeeding-related skills (Table). Word document.

## Data Availability

All data generated or analysed during this study are included in this published article [and its supplementary information files].
