# Supplementary material for: Are the doctors of the future ready to support breastfeeding? A cross-sectional study in the UK
Source: Int Breastfeed J. 2020 May 20;15:46. doi: 10.1186/s13006-020-00290-z (PMC7238622; doi:10.1186/s13006-020-00290-z)
Supplement: Supplementary file 1 — Additional file 1. Patient Information Sheet. Word document. [file 13006_2020_290_MOESM1_ESM.docx]

**Additional File 1- participant information sheet**

**Dear Participant**

**Study title:** Medical student education and the perceived role of the doctor in breastfeeding practices

We would like to invite you to take part in our research study. Before you decide we would like you to understand why the research is being done and what it would involve for you if you took part. Please ask us if there is anything that is not clear or if you would like more information.

1. **What is the purpose of the study?**

The main research questions to be addressed asks whether medical students value breastfeeding, and the role they play in supporting and promoting breastfeeding as medical professionals and whether medical students adequately prepared to support mother-infant dyads presenting with breastfeeding related problems.

1. **Who is organising and funding the research?**

Kirsty Biggs, fourth year medical student supported by Miss Heather Brown, Consultant Obstetrician Gynaecologist. This project has received no funding.

1. **Why have I been invited?**

Currently there is no published UK data on infant feeding education provided within undergraduate medicine courses. In terms of investigating how medical students perceive their role in supporting breastfeeding and to whether they feel adequately prepared to deal with breastfeeding-related problems; it is appropriate to invite medical students from all UK medical schools to partake in order to get a more representative sample. We are approaching medical students in their final two years of UK undergraduate medical courses to partake in our survey.

1. **Do I have to take part?**

No, participation is voluntary and completely anonymous.

1. **What will I have to do?**

Answer 10 multiple choice questions on an online survey.

1. **What are the possible benefits of taking part?**

The research hopes to provide insight into the current breastfeeding teaching provided by medical schools and the resultant attitudes and knowledge base of medical students. This information will inform any suggestions to change current teaching.

1. **Are there any possible disadvantages or risks of taking part?**

No

1. **What about confidentiality?**

All the information about you having taken part in this study and all information collected during the course of the research will be kept strictly confidential. It will be stored anonymously.

1. **What will happen if I don’t want to carry on with the study?**

You are free to withdraw at any time and without giving a reason. If you decide to withdraw or not join the study, there will be no negative consequences. We will also be happy to discuss with you what will happen to any data that has been collected up to the point of your withdrawal from the study

1. **What will happen to the results of the research study?**

The results of the study may be written and up and published in a scientific journal.

1. **Who has approved this study?**

This study has received ethical approval from the Brighton and Sussex Medical School Research Governance and Ethics Committee (BSMS RGEC).

**Thank you for taking the time to read this information sheet.**

**15.**  **Contact Details:** Kirsty Biggs bsms3284@uni.bsms.ac.uk
