# Supplementary material for: Are the doctors of the future ready to support breastfeeding? A cross-sectional study in the UK
Source: Int Breastfeed J. 2020 May 20;15:46. doi: 10.1186/s13006-020-00290-z (PMC7238622; doi:10.1186/s13006-020-00290-z)
Supplement: Supplementary file 2 — Additional file 2. Survey questions on SurveyMonkey. Word document. [file 13006_2020_290_MOESM2_ESM.docx]

**Additional File 2- survey questions on SurveyMonkey**

1. Which university do you attend?

                                                                                                                                                                                    

*2. Select your age category:

21-25

26-30

31-35

>36

*3. Are you male or female?

Male

Female

Prefer not to say

*4. Which of the following are known benefits of breastfeeding (tick as appropriate)

Tailor made milk containing antibodies and hormones available on demand

Reduced Necrotising enterocolitis in premature infants

Infants have reduced risk of obesity and type 2 diabetes in adulthood

Reduced infantile infections

Emotional attachment with mother through feeding

Reduced risk of ovarian and breast cancer in mother

*5. How confident would you feel with helping patients with the following?

|  | Confident | Somewhat confident | Not confident |
| --- | --- | --- | --- |
| Latching problems |  Confident |  Somewhat confident |  Not confident |
| Recognising and managing nipple problems such as mastitis and nipple thrush |  Confident |  Somewhat confident |  Not confident |
| Advising on medical reasons for supplementing breastfed infants with formula milk |  Confident |  Somewhat confident |  Not confident |

*6. In your opinion, how much impact would a daily formula milk top-up have on breastfeeding?

                                                                                         

*7. How important is your role as a doctor in supporting breastfeeding?

                                                                                         

*8. Who do you think is most influential upon a mother's infant feeding decision? (Rank from 1-6, 1= most influential)

                                                                                                                                                                                     Doctor                 

Midwife

Other healthcare professional

                                  

Her partner                

Her mother                

Other family member 

*9. Are you considering a career path in the following?

|  | Yes | No |
| --- | --- | --- |
| Obstetrics & Gynaecology | Yes | No |
| Paediatrics | Yes | No |
| General Practice |  Yes | No |

*10. Would you be interested in some teaching on breastfeeding?

Yes, Lecture on current recommendations (evidence-based)

Yes, practical training on how to support mothers

Yes to both

No thank you
