# Supplementary material for: Are the doctors of the future ready to support breastfeeding? A cross-sectional study in the UK
Source: Int Breastfeed J. 2020 May 20;15:46. doi: 10.1186/s13006-020-00290-z (PMC7238622; doi:10.1186/s13006-020-00290-z)
Supplement: Supplementary file 3 — Additional file 3. Medical school curriculum details. Word document. [file 13006_2020_290_MOESM3_ESM.docx]

**Additional File 3**

Medical school curriculum details

| **University** | **No. student responses** | **Formal clinical teaching** | **Compulsory to attend** | **Year group** | **Details** |
| --- | --- | --- | --- | --- | --- |
| 1 | pre-clinical | No | No | 2 | One lecture on the physiology of lactation including a section on breastfeeding. All lectures are optional, but content included in summative exams |
| 2 | 88 | No | Yes | 1,2,4 | The following sessions include breastfeeding but are not exclusively devoted to it:  Year 1: Lectures - developmental/ health benefits of breastfeeding, psychological aspects, nutrition. Family study (visiting family with a new baby over the year).  Year 2: Lecture - physiology of breastfeeding  Year 4: Paediatric and obstetric rotations (ad-hoc), one-hour lecture on breastfeeding in general practice. |
| 3 | 1 | No | Yes | 2,4 | Year 2: Case based learning combining lecture and community learning (approximately 4 hours).  Year 4: Further learning opportunities within specialty block |
| 4 | 24 | No | Yes | 4 | Year 4: Obstetrics and gynaecology placement taught by midwives and consultants on the ward, small group teaching (highlighting the evidence base), self-directed learning and through case based. |
| 5 | 14 | Yes | Yes | 1 | Year 1: Lecture, problem-based learning and interactive session with breast feeding mums. There may be other teaching in later years. |
| 6 | 12 | No | No | 5 | Year 5: Part of lecture teaching session about the Puerperium in the obstetrics and gynaecology lecture course. Lectures are optional. |
| 7 | 10 | No | Yes | 2 | Year 2: Part of nutrition course learning objective 'Breast milk: explain the benefits of breast milk |
| 8 | Declined | No | Yes | 2,4 | Year 2: Online module on prescribing for breastfeeding women, lecture: milk production and breastfeeding  Year 4: Lecture on postnatal period includes some information on the benefits of breast vs bottle feeding during obstetrics and gynaecology rotation. |
| 9 | 0 | Yes | Yes |  | Lecture and Small group work, during obstetrics and gynaecology clinical teaching |
| 10 | 17 | No | No | 4 | Year 4: Postnatal ward time spent with midwives and post-natal patients, opportunities to attend dedicated breastfeeding midwifery led clinics. |
| 11 | 28 | No | Yes | 4 | Year 4: Lecture, portfolio case, community midwife attachment (2 days) |
| 12 | 1 | Yes | Yes | 1,2,4 | Year 1 and 2: Lectures covering basic science, pros and cons and clinical issues  Year 4: Clinical small group tutorials during obstetrics and gynaecology rotation (1 hour) and paediatrics rotation (1 hour). All students spend a day on home visits with community midwives. |
| 13 | 3 | No | Yes | 4 | Year 4: Lecture and seminar |
| 14 | 2 | No | Yes | 1 | Year 1: Lecture given by specialist midwife |
| 15 | 2 | No | Yes | 1,3,4 | Year 1 Lecture (one hour) embedded within a three-week problem-based learning case.  Year 3+4: Clinical placements may include breastfeeding.  Problem based learning and placements are compulsory. Lectures are recommended (each student must attend more than 70% per annum). |
| 16 | 23 | No | Yes | 3 | Year 3: Lecture (one hour) and e-learning modules. Planning to run a 4-week student select component (SSC) on infant feeding |
| 17 | pre-clinical | No | Yes | 1 | Year 1: There is no single session on lactation/breastfeeding; teaching takes place in the context of 'Life cycle' lectures and a project in which students visit a family with a new baby or young child in the family (the Family Project).  The relevant learning outcomes are as follows:  1) Describe the histological changes in the breast during lactation  2) Describe the mechanism of milk production and release  3) Describe the nutritional properties of breast milk and their differences from artificial milks  4) Describe the factors that influence maternal feeding decisions |
| 18 | 21 | No | Yes | 1,2,4 | Lecture based teaching on lactation physiology and family study. Session with postnatal midwifery team during obstetrics and gynaecology rotation. |
| 19 | Declined | No | No | n/a | No teaching provided |
| 20 | 32 |  |  |  |  |
| 21 | 30 |  |  |  |  |
| 22 | 45 | No | Yes | 1 | Scientific/immune benefits covered in basic science lectures Practical issues covered in GP, obstetrics and gynaecology & in community GP tutorials |
| 23 | 2 | Yes | Yes | 3 | Midwife led small group teaching sessions during obstetrics block  Lecture/seminar: Physiology of pregnancy |
| 24 | 0 | No | Yes |  | Year 1-3: unknown  Clinical years: Paediatric seminar on neonatal complications and how breastmilk reduces the risk of necrotising enterocolitis. Obstetrics and gynaecology tutorial on post-partum problems includes some information on breastfeeding. No specific breastfeeding education.  Learning objectives include:   1. students must be aware of the ‘importance of breastfeeding’ 2. ‘breast and formula feeding – have a good understanding of both’ |
| 25 | 10 |  |  |  |  |
| 26 | 4 | Yes | Yes | 3 | Small group teaching led by practice educator midwife |
| 27 | Declined |  |  |  |  |
| 28 | 36 |  |  |  |  |
| 29 | 0 | No | Yes | 3,4,5 | Year 3: Physiology and anatomy teaching  Year 4- Tutorial in Obstetrics and Gynaecology rotation,  Year 4 or 5: GP placement tutorial/case-based discussion.  Features in the OSCE assessment bank for degree exams. |
| 30 | Declined | No | Yes | 3,5 | Obstetrics and Gynaecology rotation: session on the postnatal ward with midwife |
| 31 | Declined |  |  |  |  |
| 32 | 6 | No | Yes | 5 | Lecture: 'the puerperium' includes information in breastfeeding during obstetrics and gynaecology introductory teaching week.  Ad-hoc clinical: students spend around 4-5 days on the labour ward, with midwives, supporting women in labour, delivery and the initial neonatal period; this may include observing the initial feed and discussion between the midwife and the woman regarding breastfeeding advice. |
