# Supplementary material for: Are the doctors of the future ready to support breastfeeding? A cross-sectional study in the UK
Source: Int Breastfeed J. 2020 May 20;15:46. doi: 10.1186/s13006-020-00290-z (PMC7238622; doi:10.1186/s13006-020-00290-z)
Supplement: Supplementary file 4 — Additional file 4. Table of medical students’ ability to correctly identify the benefits of breastfeeding (Table). Word document. [file 13006_2020_290_MOESM4_ESM.docx]

**Additional File 4**

Medical students’ ability to correctly identify the benefits of breastfeeding:

| Knowledge on the benefits of BF | n=411 (%) |
| --- | --- |
| Emotional attachment | 400 (97) |
| Tailor made antibodies and hormones | 377 (92) |
| Reduces Infantile infection | 368 (90) |
| Protective of maternal cancer | 320 (78) |
| Reduces obesity and T2DM in later life | 317 (77) |
| Reduces risk of NEC | 284 (69) |
| Reduced environment impact | 253 (62) |
