# Supplementary material for: Are the doctors of the future ready to support breastfeeding? A cross-sectional study in the UK
Source: Int Breastfeed J. 2020 May 20;15:46. doi: 10.1186/s13006-020-00290-z (PMC7238622; doi:10.1186/s13006-020-00290-z)
Supplement: Supplementary file 5 — Additional file 5. Medical students self-rated confidence in clinical skills in relation to teaching provided by medical school (Table). Word document. [file 13006_2020_290_MOESM5_ESM.docx]

**Additional File 5**

Confidence in clinical skills vs teaching received

| **Medical students self-rated confidence in clinical skills in relation to teaching provided by medical school n= 411 (%)** | | | | | |
| --- | --- | --- | --- | --- | --- |
|  |  |  |  |  |  |
|  | No of responders receiving teaching | Confident | Somewhat confident | Not confident | P Value |
| **Latching** |  |  |  |  |  |
| Received compulsory teaching | 274 | 10 (4) | 50 (18) | 214 (78) | 0.25 |
| Received seminar/small group teaching | 90 | 3 (3) | 19 (21) | 68 (76) | 0.89 |
| Received formal clinical teaching | 21 | 1 (5) | 4 (19) | 16 (76) | 0.96 |
| **Nipple Rx** |  |  |  |  |  |
| Received compulsory teaching | 274 | 37 (14) | 130 (47) | 107 (39) | 0.81 |
| Received seminar/small group teaching | 90 | 18 (20) | 49 (54) | 23 (26) | **<0.01** |
| Not received seminar/small group | 213 | 23 (11) | 93 (44) | 97 (46) |  |
| Received formal clinical teaching | 21 | 0 (0) | 15 (71) | 6 (29) | **0.04** |
| Not received formal clinical teaching | 282 | 41 (15) | 127 (45) | 114 (40) |  |
| **Formula Use** |  |  |  |  |  |
| Received compulsory teaching | 274 | 32 (12) | 95 (35) | 147 (54) | 0.09 |
| Received seminar/small group teaching | 90 | 12 (13) | 32 (36) | 46 (51) | 0.75 |
| Received formal clinical teaching | 21 | 1 (5) | 10 (48) | 10 (48) | 0.43 |
| Lecture teaching was not analysed as the teaching provided in lectures was not of a clinical skill relevance. Ad-hoc clinical teaching was excluded from analysis as it could not be determined whether medical student had any clinical exposure or teaching. | | | | | |
