# Supplementary material for: Are the doctors of the future ready to support breastfeeding? A cross-sectional study in the UK
Source: Int Breastfeed J. 2020 May 20;15:46. doi: 10.1186/s13006-020-00290-z (PMC7238622; doi:10.1186/s13006-020-00290-z)
Supplement: Supplementary file 6 — Additional file 6. Ranking of influential figures according to interest in obstetrics and gynaecology/paediatrics/general practice (Table). Word document. [file 13006_2020_290_MOESM6_ESM.docx]

**Additional File 6**

Ranking of influential figures according to interest in obstetrics and gynaecology/paediatrics/general practice.

| Influential Figure | Mean ranking (1-6) | Ranking of mean score | Interest in O+G/ P/GP | Ranking of mean score | No interest | Ranking of mean score |
| --- | --- | --- | --- | --- | --- | --- |
| Partner | 2.39 | 1 | 2.38 | 1 | 2.4 | 1 |
| Midwife | 2.6 | 2 | 2.55 | 2 | 2.83 | 2 |
| Mother | 3.01 | 3 | 2.97 | 3 | 3.2 | 3 |
| Doctor | 3.76 | 4 | 3.78 | 4 | 3.69 | 4 |
| Other family | 4.4 | 5 | 4.43 | 5 | 4.24 | 5 |
| Other HCP | 4.8 | 6 | 4.83 | 6 | 4.64 | 6 |
