# Supplementary material for: Are the doctors of the future ready to support breastfeeding? A cross-sectional study in the UK
Source: Int Breastfeed J. 2020 May 20;15:46. doi: 10.1186/s13006-020-00290-z (PMC7238622; doi:10.1186/s13006-020-00290-z)
Supplement: Supplementary file 7 — Additional file 7. Influences upon medical students’ perceived importance of the doctor’s role in Breastfeeding support (Table). Word document. [file 13006_2020_290_MOESM7_ESM.docx]

**Additional File 7**

Influences upon medical students' perceived importance of the doctor's role in BF support

| **Influences upon medical students' perceived importance of the doctor's role in BF support n=411 (%)** | | | | | |
| --- | --- | --- | --- | --- | --- |
|  |  |  |  |  |  |
|  | No of responders | Very important | Quite important | Not important | P Value |
| Age (years) |  |  |  |  | **0.04** |
| 21-25 | 347 | 157 (45) | 170 (49) | 20 (6) |  |
| 26-30 | 46 | 17 (37) | 25 (54) | 4 (9) |  |
| 31-36 | 9 | 3 (33) | 3 (33) | 3 (33) |  |
| >36 | 9 | 5 (56) | 4 (44) | 0 (0) |  |
| Male | 116 | 46 (40) | 62 (53) | 8 (7) | 0.48 |
| Female | 294 | 136 (46) | 140(48) | 18 (6) |  |
| Interest in O+G/Paeds/GP | 336 | 160 (48) | 154 (46) | 22 (7) | **0.01** |
| Not interested | 75 | 22 (29) | 48 (64) | 5 (7) |  |
| Lectures | 258 | 110 (43) | 129 (50) | 19 (7) | 0.43 |
| No lectures | 45 | 21 (47) | 23 (51) | 1 (2) |  |
| Seminars | 90 | 41 (46) | 43 (48) | 6 (7) | 0.86 |
| No seminars | 213 | 90 (42) | 109 (51) | 14 (7) |  |
| Formal clinical teaching | 21 | 11 (52) | 10 (48) | 0 (0) | 0.38 |
| No formal clinical teaching | 282 | 120 (43) | 142 (50) | 20 (7) |  |
| Ad-hoc clinical | 200 | 88 (44) | 97 (49) | 15 (8) | 0.57 |
| No ad-hoc | 103 | 43 (42) | 55 (53) | 5 (5) |  |
