# Supplementary material for: Are the doctors of the future ready to support breastfeeding? A cross-sectional study in the UK
Source: Int Breastfeed J. 2020 May 20;15:46. doi: 10.1186/s13006-020-00290-z (PMC7238622; doi:10.1186/s13006-020-00290-z)
Supplement: Supplementary file 8 — Additional file 8. Interest in further teaching in medical schools according to medical students’ confidence at performing breastfeeding-related skills (Table). Word document. [file 13006_2020_290_MOESM8_ESM.docx]

**Additional File 8**

Interest in further teaching in medical schools according to medical students’ confidence at performing breastfeeding-related skills.

| **Requests for further teaching in medical school, according to clinical confidence** | | | | |
| --- | --- | --- | --- | --- |
|  | Confident | Somewhat Confident | Not Confident | P-value |
| **Latching confidence** |  |  |  | 0.13 |
| Wanted teaching | 3.2% | 20.7% | 76.1% |  |
| Did not want teaching | 5.9% | 29.4% | 64.7% |  |
| **Nipple problems confidence** |  |  |  | 0.63 |
| Wanted teaching | 12.8% | 59.0% | 28.2% |  |
| No teaching | 16.8% | 42.6% | 40.5% |  |
| **Formula use confidence** |  |  |  | 0.28 |
| Wanted teaching | 14.8% | 39.2% | 57.9% |  |
| Did not want teaching | 8.8% | 47.1% | 44.1% |  |
|  |  |  |  |  |
| **Requests for combined teaching (practical + lectures), according to clinical confidence** | | | | |
|  | Confident | Somewhat Confident | Not Confident | P-value |
| **Latching confidence** |  |  |  | 0.22 |
| Wanted teaching | 3.4% | 19.5% | 77.2% |  |
| Did not want teaching | 3.5% | 25.0% | 71.5% |  |
| **Nipple problems confidence** |  |  |  | **<0.01** |
| Wanted teaching | 15.0% | 41.2% | 43.8% |  |
| Did not want teaching | 22.8% | 54.2% | 27.8% |  |
| **Formula use confidence** |  |  |  | **0.01** |
| Wanted teaching | 11.6% | 32.6% | 55.8% |  |
| Did not want teaching | 36.8% | 42.4% | 42.4% |  |
